# Mapping HRV in sports science: from monitoring to machine learning

**DOI:** 10.3389/fspor.2025.1714962

**Published:** 2026-01-12

**Authors:** Zhongyang Wang, Jing Hu, Wenbing Yu

**Affiliations:** Teaching Center of Fundamental Courses, Ocean University of China, Qingdao, China

**Keywords:** bibliometrics, CiteSpace, heart rate variability (HRV), sports science, VOSviewer

## Abstract

**Background:**

Heart Rate Variability (HRV) is a crucial non-invasive marker of autonomic nervous system function, extensively applied in sports science for monitoring training load, fatigue, recovery, and performance optimization. The rapid expansion and diversification of HRV research necessitate a comprehensive bibliometric analysis to map the knowledge structure and emerging trends.

**Objective:**

This study employed innovative bibliometric visualization to quantitatively analyze the literature landscape, research hotspots, and evolutionary trends in HRV applications within sports from 2010 to 2025. It aimed to identify key contributors, delineate major research themes, uncover nascent directions, and identify emerging research trajectories.

**Method:**

Utilizing CiteSpace 6.3.R1 and VOSviewer, we conducted a comprehensive visual analysis of 1,660 articles retrieved from the Web of Science Core Collection and Scopus databases. This study performed co-authorship, co-citation, keyword co-occurrence, cluster analysis, and burst detection to unveil publication trends, collaborative networks, influential works, core authors, research hotspots, and emerging trends.

**Results:**

Publication volume showed a significant growth trend, peaking in 2022 with 209 articles. The USA and Brazil were the most productive countries, with the University of São Paulo leading institutionally. Document co-citation analysis identified five major research hotspots: Athlete Monitoring, Biofeedback, Sport-related Concussion, Anxiety, and Endurance Exercise. Keyword burst analysis revealed three dominant future trends: “Sleep,” “Machine Learning,” and “Anxiety”.

**Conclusion:**

This bibliometric analysis delineates the evolution of HRV research in sports, confirming established domains while highlighting the importance of HRV's role in concussion management and psychological assessment. Critically, it highlights the field's evolving trajectory, emphasizing the growing integration of sleep interactions, machine learning-driven personalization, and the dynamics of HRV and anxiety. These findings provide a structured roadmap for future research and practical applications.

## 1 Introduction

Heart Rate Variability (HRV), a non-invasive quantitative measure of autonomic nervous system (ANS) function, exhibits alterations closely associated with physiological stress, recovery, training adaptation, and overall health status (1, 2). In sports science, HRV has become a critical tool for monitoring training load, evaluating fatigue and recovery, preventing overtraining, and optimizing athletic performance (3, 4). Analysis of HRV provides insights into athletes’ immediate and long-term responses to training stimuli and the adaptive potential of their ANS (5, 6), offering a vital basis for individualized training guidance, recovery strategy adjustment, and mitigating the risk of maladaptation (7, 8). However, the precise interpretation and effective application of HRV are susceptible to interference from multiple factors (9, 10), and optimal measurement protocols, analytical procedures, and individualized thresholds require further investigation (3, 11).

Given the rapid growth in the volume of related literature and the diversification of research topics, a bibliometric analysis of the field's knowledge structure is particularly necessary. Bibliometric analysis, as an objective and rigorous quantitative research method, can effectively reveal structural patterns and developmental trends within large volumes of literature data (12). Using visualization tools such as CiteSpace, this method can identify core research directions, key publications, major institutions, and leading scholars, as well as track research hotspots and frontier trends (13, 14). This assists researchers in grasping the field's overall development, identifying knowledge gaps, and providing guidance for future research planning.

Therefore, this study aims to utilize CiteSpace software to conduct a visual analysis of 1,660 articles on the application of HRV in the sports field, retrieved from the Web of Science and Scopus databases for the period of 2010 to 2025. The analysis will encompass co-authorship networks, document co-citation patterns, keyword co-occurrence, cluster analysis, and burst detection. The objective is to systematically map the knowledge landscape of the field, identify core research themes, reveal emerging trends, and map emerging research frontiers. This study is expected to provide a structured, macroscopic overview for researchers, coaches, and practitioners in the field, offering a valuable reference for subsequent scientific research and practical applications.

## 2 Materials and methods

### 2.1 Data source and search strategy

The systematic review protocol employed in this study was adapted from the Preferred Reporting Items for Systematic Reviews and Meta-Analyses (PRISMA) 2020 guidelines to suit the specific requirements of a bibliometric analysis. While PRISMA is primarily designed for systematic reviews of clinical trials, its principles of transparency and reproducibility were applied to our data collection, screening, and inclusion processes.

**Figure 1:**
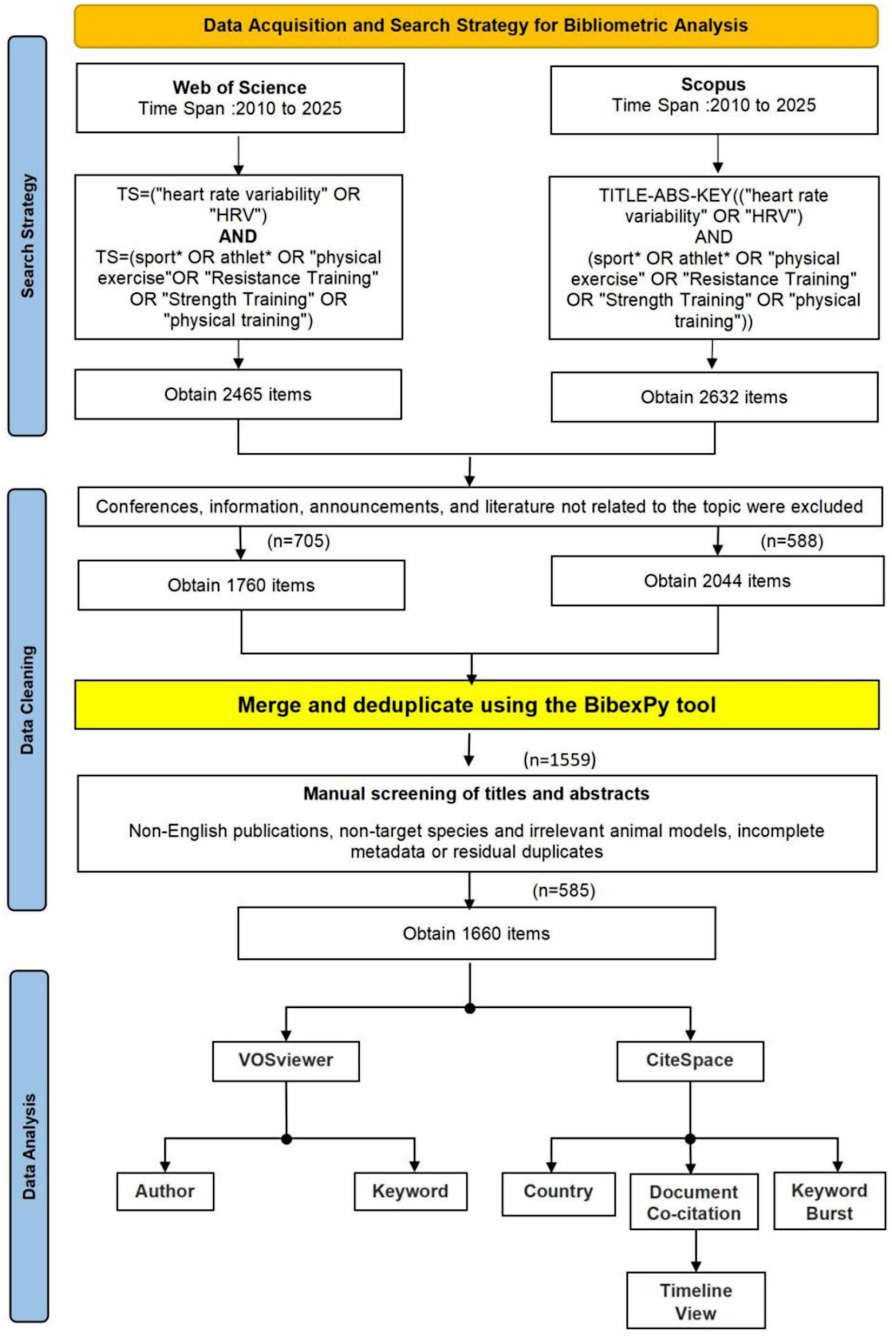
Data retrieval flow chart.

A systematic literature search was conducted on August 20, 2025, in the Web of Science (WOS) Core Collection and Scopus databases, covering the period from 2010 to 2025. As shown in Figure 1, using specific search strategies (WOS: TS = (“heart rate variability” OR “HRV”) AND TS = (sport* OR athlet* OR “physical exercise” OR “Resistance Training” OR “Strength Training” OR “physical training”); Scopus: TITLE-ABS-KEY[(“heart rate variability” OR “HRV”) AND (sport* OR athlet* OR “physical exercise” OR “Resistance Training” OR “Strength Training” OR “physical training”)]), an initial total of 2,465 (WOS) and 2,632 (Scopus) articles were retrieved. After initially excluding conferences and irrelevant studies, the datasets were harmonized using the BibexPy tool. This process utilized an ‘Intelligent Merging’ mechanism that identified duplicates via DOI matching or normalized Title/Year keys. Instead of simply discarding redundant entries, conflicts were resolved by consolidating complementary metadata (e.g., merging keyword lists from both databases) to enrich the dataset (15). Subsequently, a rigorous manual screening of titles and abstracts was conducted to remove non-English publications, non-target species, and records with incomplete metadata, resulting in a final selection of 1,660 articles. Finally, VOSviewer was employed for co-occurrence analysis of authors, keywords, and countries, while CiteSpace was used for document co-citation analysis, keyword burst detection, and timeline visualization.

### 2.2 Visual analysis method

This study employed CiteSpace 6.3.R1 software, utilizing the retrieved English literature related to the application of HRV in the sports field as the research data. Knowledge graph analysis was conducted on elements such as authors, countries, institutions, keywords, and references from the literature. This approach aimed to identify prolific authors, countries, and institutions in the field of HRV in sports since 2010, explore research hotspots, and analyze research trends in this domain.

### 2.3 Parameter settings

The time span for data import was set from 2010 to 2025, with the time slice (Years Per Slice) configured to one year to facilitate a more fine-grained annual tracking of the field's research progress. To ensure the analysis focused on the most influential research outputs each year, the node selection criterion was uniformly set to “Top N per slice=100.” This means that within each annual slice, only the top 100 records (e.g., articles, authors, keywords) based on citation or occurrence frequency were extracted for analysis. Furthermore, the Look Back Years (LBY) parameter was set to ensure that the calculation of co-occurrence or co-citation strength focused on recent and more active academic linkages. Specific parameter settings were configured to optimize the structural clarity of the visualization maps. For CiteSpace, the time span was set to 2010–2025 with a time slice of 1 year. The node selection criterion was generally set to ‘Top N’ with *N* = 100 per slice (e.g., for document co-citation), with the exception of the country collaboration analysis, which utilized the g-index (k = 25). Network links were configured with a Link Retaining Factor (LRF) of 2.0, a Look Back Years (LBY) setting of 5, and an e value of 1.0. No pruning strategy was applied. Detailed parameter configurations are summarized in Table 1.

**Table 1:** Key parameter settings for bibliometric analysis.

Software	Parameter category	Configuration/value
CiteSpace (v6.3.R1)	Time slicing	2010–2025 (Slice Length = 1 year)
	Node selection	Top N per slice (*N* = 100)
	Link assessment	Link Retaining Factor (LRF) = 2.0
		Look Back Years (LBY) = 5
		Max Links per Node (L/N) = 5
		Link Strength (e) = 1.0
	Network pruning	None (No pruning applied)
VOSviewer	Analysis type	Co-occurrence (Keywords)/Co-authorship
	Counting method	Full Counting
	Min. occurrences	30 (Keywords), 5(Authors)

### 2.4 Data analysis and judgment criteria

In the visual knowledge graphs, the size of a node represents its frequency of occurrence, and the thickness of the lines between nodes indicates the strength of their connection. A node with centrality greater than 0.1 is marked in purple, signifying its higher importance. In the knowledge graph, a modularity Q value >0.3 indicates that the cluster structure is significant. When the mean silhouette S value is >0.5, the clustering result is considered reasonable, and an S value >0.7 indicates a very significant clustering result. Cluster analysis of document co-citations can explore the hotspots and frontiers of the field. In the burst detection graph for cited references, red segments highlight the burst period of citations in corresponding years, which can be used to identify emerging research trends. Cluster analysis of keywords can reflect the research hotspots in the field.

## 3 Results

### 3.1 Overall characteristics of publications

Changes in publication volume can reflect the research level and development stage of a field (16). To a certain extent, it serves as an important source for gauging dynamic development trends, the degree of attention received, and identifying potential research trajectories in the area.

**Figure 2:**
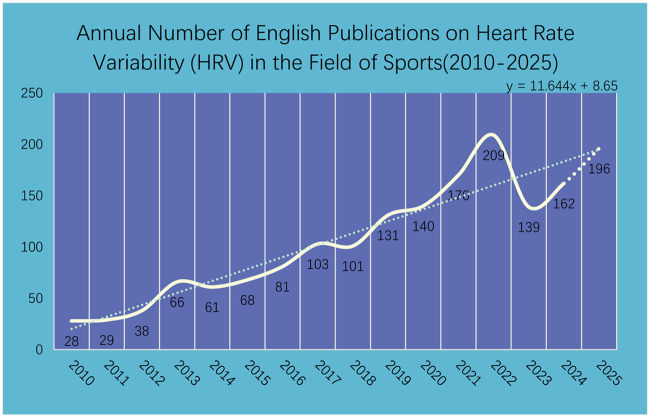
Annual number of English publications on heart rate variability (HRV) in the field of sports (2010–2025).

The analysis of publication volume from 2010 to early 2025 indicates that this research field has shown a significant growth trend (Figure 2). Research output demonstrated characteristics of phased accelerated growth, particularly after 2012 and 2018. The annual number of publications consistently surpassed one hundred from 2019 onwards, reaching a peak of 209 articles in 2022. Although the publication volume showed a slight decline from the peak in 2023 (141 articles), the 160 articles published in 2024 indicate that research enthusiasm remains at a high level. As noted in the methods, the data for 2025 is partial; 137 relevant articles were published within this retrieval window (up to August 20), signaling that robust research interest is continuing into the current year.

### 3.2 Analysis of country

**Figure 3:**
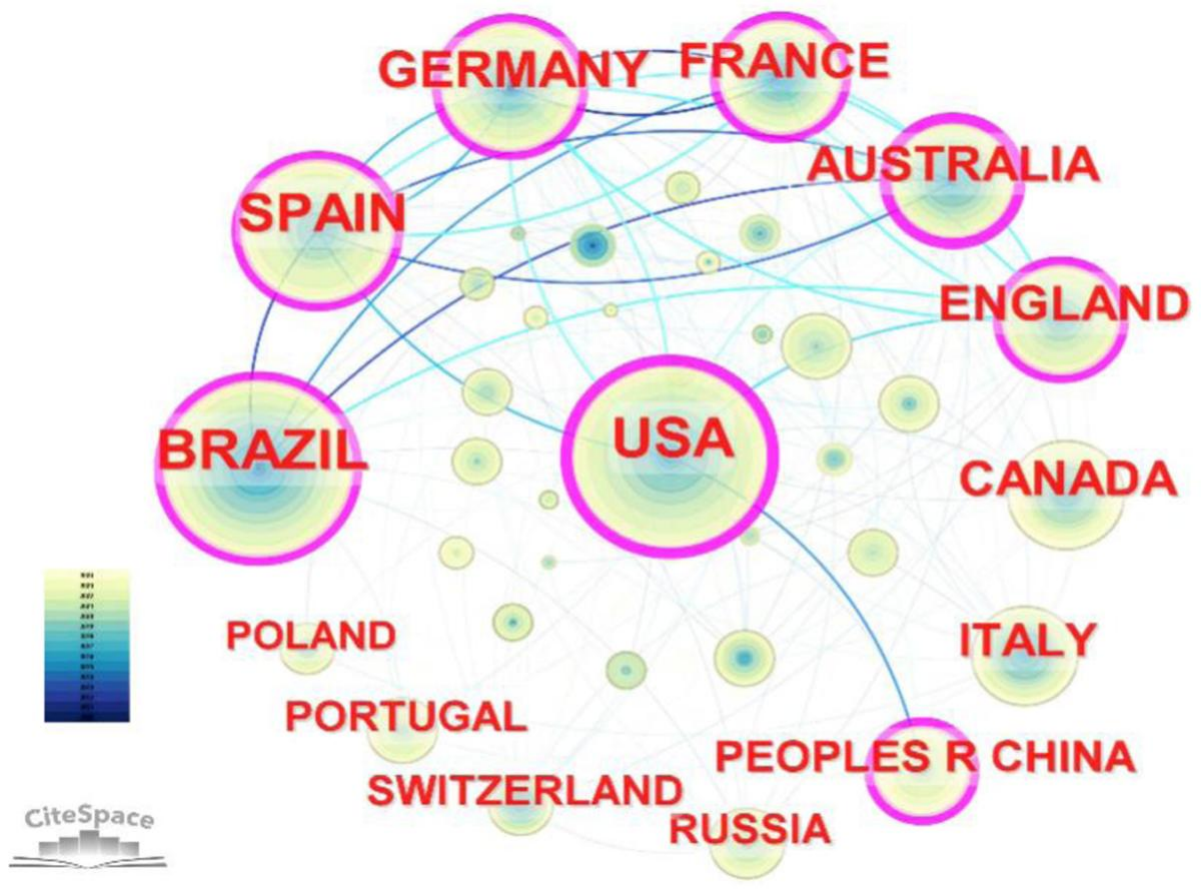
Publication volume by country on heart rate variability (HRV) in the field of sports and exercise.

As illustrated in Figure 3, the global collaborative network (76 nodes, 396 links, density=0.1389) reveals a highly interconnected yet stratified landscape. The United States (*N* = 216) and Brazil (*N* = 213) emerged as the two dominant poles, followed by Spain (*N* = 150), Germany (*N* = 119), and France (*N* = 117). The network visualization demonstrates a clear “hub-and-spoke” pattern centered on the USA, which radiates dense collaborative ties to Europe, Brazil, and Asia. Notably, Australia exhibited the highest betweenness centrality (0.31), acting as a critical bridge in the network despite a lower total publication volume compared to the USA (0.25) and Brazil (0.18). This suggests that while Brazil is a prolific producer, its collaborations are more internally concentrated, whereas Australia serves as a key connector between distinct research clusters. China (*N* = 70) presents a unique profile. Its node displays a prominent red inner ring (citation burst), signaling a recent and significant surge in research activity. However, a structural analysis of its links reveals that China's international collaborations are heavily anchored to the USA, with relatively weak connectivity to European or Australian networks.

This observed geographical distribution is underpinned by distinct national research ecosystems. The leadership of the USA is largely sustained by a robust federal funding structure (e.g., NIH grants) and a highly commercialized sports technology market, which drives continuous validation studies of wearable devices. In contrast, Brazil's prominence—an outlier for the Global South—is closely linked to state-sponsored internationalization initiatives, most notably the “Science Without Borders” program. This policy facilitated massive scholar mobility, effectively integrating Brazilian researchers into elite global networks. Meanwhile, Spain's output is incentivized by national evaluation systems (e.g., the “Sexenio”) that reward high-impact publications, fostering a culture of prolific output.

### 3.3 Analysis of institution

According to the provided data on institutional publication volume, Brazil demonstrates particular prominence in this research field. As presented in Table 2, the University of São Paulo (USP) leads globally with 48 articles, followed by the State University of Londrina (UEL) (31 articles) and São Paulo State University (UNESP) (29 articles). The University of Milan (Italy) ranks fourth with 28 articles, while the German Sport University Cologne (Germany) ties for fifth place (26 articles). Beyond publication counts, these leading institutions exhibit distinct research focal points that drive the field's evolution:

**Table 2:** Top 10 institutions in research on heart rate variability in the field of sports.

Rank	Institution	Country	Counts
1	University of São Paulo	Brazil	48
2	State University of Londrina	Brazil	31
3	São Paulo State University	Brazil	29
4	University of Milan	Italy	28
5	German Sport University Cologne	Germany	26
6	James Cook University	Australia	26
7	University of Alabama	United States	26
8	European University of Madrid	Spain	25
9	Georgia Southern University	United States	19
10	University of Granada	Spain	19

University of São Paulo (USP): Emphasizes the application of HRV in assessing cardiovascular autonomic regulation following aerobic training, focusing on the physiological mechanisms of autonomic adaptations and their implications for cardiovascular health and physical performance (17–19).

State University of Londrina (UEL) & São Paulo State University (UNESP): These centers prioritize team sports (e.g., soccer and basketball), focusing on the sensitivity of ultra-short-term HRV metrics (e.g., lnRMSSD) to training stimuli. Their work is pivotal in guiding load periodization and fatigue management strategies (20, 21).

University of Milan: Investigates the critical link between HRV and overtraining syndrome, particularly in elite soccer, exploring HRV's potential as a robust biomarker for performance readiness and recovery status (22, 23).

German Sport University Cologne: Distinguishes itself by applying HRV biofeedback to enhance psychophysiological performance and evaluating the reliability of HRV thresholds in diverse domains ranging from rowing to esports (24, 25).

### 3.4 Analysis of authors and co-cited authors

Nakamura, Fabio Y. ranks first in terms of publication volume with 33 articles, followed by Flatt, Andrew A. (29 articles) and Esco, Michael R. (25 articles), who constitute the most prolific group of scholars. Regarding the co-citation frequency of literature, CAMM AJ (co-cited 477 times), BUCHHEIT M (co-cited 363 times), and PLEWS DJ (co-cited 239 times) are scholars with high academic influence in this field, whose work is widely co-cited.

**Figure 4:**
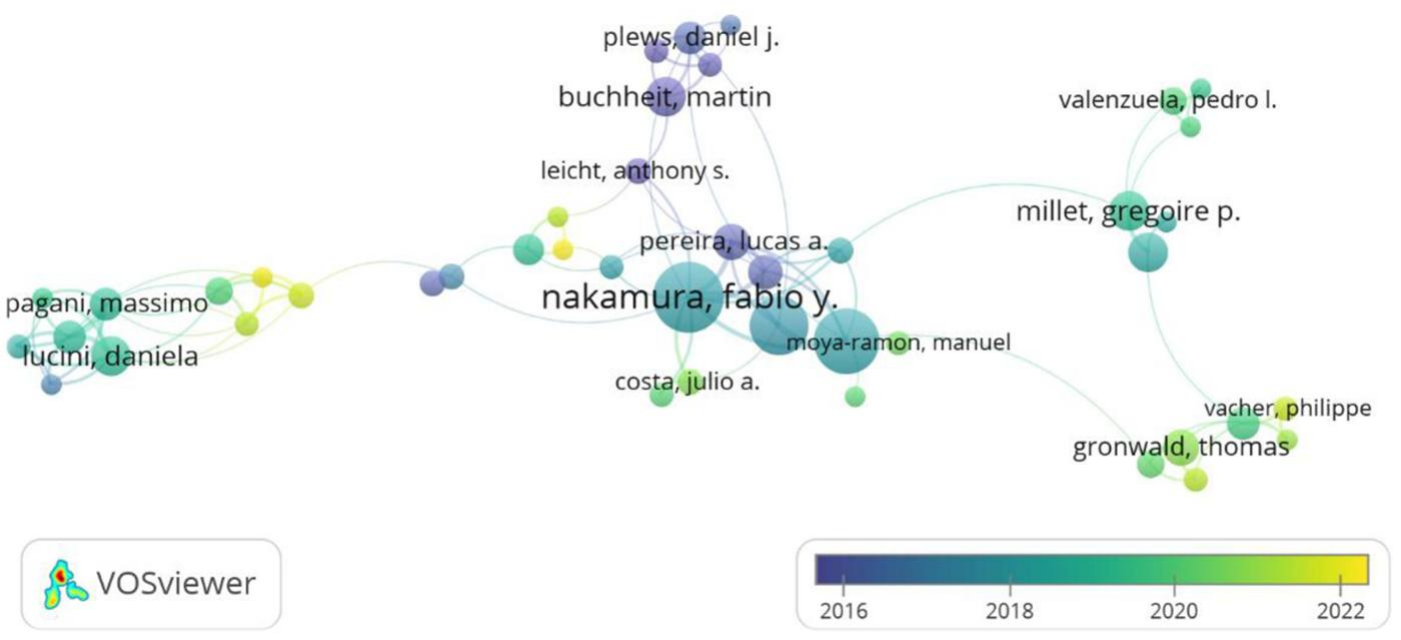
Visualization of author collaboration networks.

As shown in Figure 4; Table 3, a comparison of the two lists reveals that some scholars (such as Flatt, Schmitt, Plews, among others) possess both high productivity and high impact. The VOSviewer network map visually displays the collaboration relationships among authors, forming several main collaborative clusters centered around figures like Nakamura, Buchheit, Millet, and Pagani. The color of the nodes in the map (ranging from blue-purple to yellow, indicating earlier to more recent times) further reveals that most core authors and their teams have remained active in recent years (represented by greener and yellowish nodes in the map).

**Table 3:** Most prolific authors in heart rate variability research within the sports field.

Rank	Author	Documents	Rank	Co-cited authors	Counts
1	NAKAMURA, FABIO Y.	33	1	CAMM AJ	477
2	FLATT, ANDREW A.	29	2	BUCHHEIT M	363
3	ESCO, MICHAEL R.	25	3	PLEWS DJ	239
4	LABORDE, SYLVAIN	15	4	HOPKINS WG	185
5	NEARY, J. PATRICK	14	5	TARVAINEN MP	163
6	BUCHHEIT, MARTIN	13	6	THAYER JF	155
7	SCHMITT, LAURENT	13	7	COHEN J	152
8	MILLET, GREGOIRE P.	13	8	AUBERT AE	145
9	LUCINI, DANIELA	13	9	SHAFFER F	136
10	PEREIRA, LUCAS A.	12	10	KIVINIEMI AM	133
11	GRONWALD, THOMAS	12	11	FLATT AA	129
12	SINGH, JYOTPAL	12	12	FOSTER C	115
13	LOTURCO, IRINEU	11	13	STANLEY J	110
14	VILLAFAINA, SANTOS	11	14	TULPPO MP	108
15	PLEWS, DANIEL J.	10	15	MOUROT L	102
16	MOUROT, LAURENT	10	16	BILLMAN GE	98
17	PAGANI, MASSIMO	10	17	AL HADDADH	95
18	MALACARNE, MARA	10	18	SCHMITT L	95
19	KYROLAINEN, HEIKKI	9	19	IELLAMO F	93
20	VANDERLEI, LUIZ CARLOS M.	9	20	LABORDE S	89

Collectively, these three scholars have played a pivotal role in transitioning HRV monitoring from clinical laboratory settings to practical, field-based applications. Nakamura is a leading advocate for ultra-short-term HRV metrics (e.g., 1-minute recordings) to facilitate rapid monitoring in team sports (20). Complementing this, Flatt has pioneered the validation of smartphone applications for daily monitoring in collegiate athletes (26). Meanwhile, Esco has provided critical methodological validation, ensuring the agreement between portable devices (e.g., PPG sensors) and gold-standard ECG measurements (27).

### 3.5 Cluster analysis of document Co-citation

Co-citation analysis is a method used to identify the relationships in which two or more documents are cited together by other documents, which helps to discover important works and research hotspots within a specific field (28). This method can reveal clusters of highly cited documents, providing a concise overview of research hotspots in the field of heart rate variability in sports, and deepening the understanding of key issues. Keyword clustering analysis, as a text mining technique, aims to group keywords from a set of documents based on their semantic similarity or co-occurrence, thereby uncovering potential associations. By analyzing the co-occurrence frequency and centrality of specific keywords within a domain, researchers can identify the research hotspots represented by these keywords (29). However, relying solely on co-citation analysis may not fully reflect research hotspots, as newly published papers tend to have lower citation frequencies. Therefore, a comprehensive approach that integrates co-citation analysis and keyword clustering analysis, supplemented by manual summarization, can provide a more thorough understanding of research hotspots in this field.

In the network, a high modularity Q value (>0.3) reflects that the co-citation network possesses a clear modular structure, meaning that documents form distinct groups based on their co-citation relationships. When the mean silhouette S value is >0.7, it indicates that the clustering result is very significant, signifying that the co-cited documents within each cluster are closely related, represent a relatively consistent knowledge base, and that the research themes represented by different clusters are significantly distinct. The map generated in this study (Q = 0.8935, S = 0.9612) effectively reveals the intrinsic knowledge structure of research on HRV in the sports domain (13).

**Figure 5:**
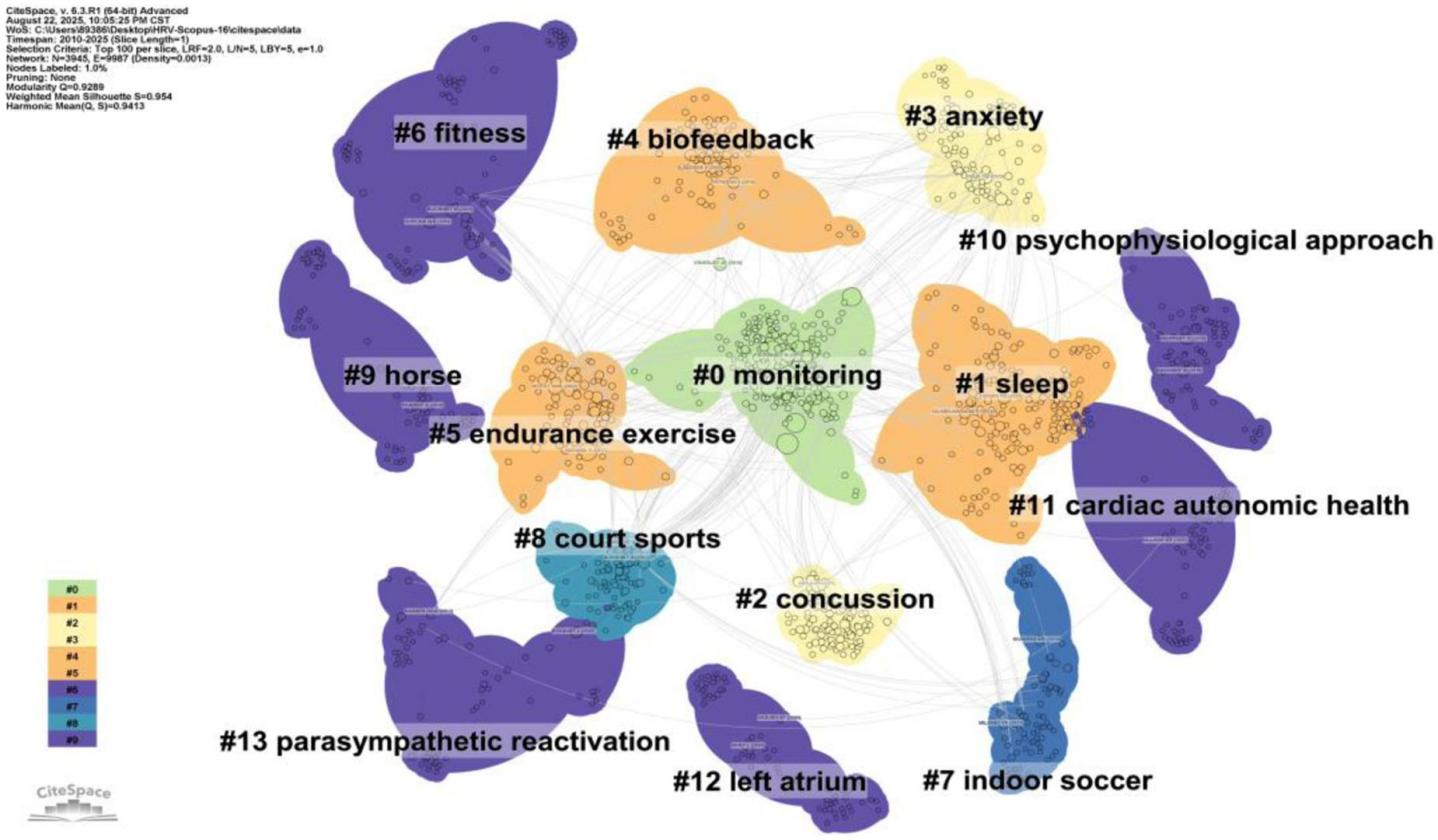
Visualization of the document Co-citation network clusters. The network comprises 15 clusters labeled by the Log-Likelihood Ratio (LLR) algorithm. To facilitate interpretation, these are grouped into five thematic domains: **(1) Athlete Monitoring** (#0 monitoring, #7 indoor soccer, #8 court sports); **(2) Recovery & Health** (#1 sleep, #6 fitness, #11 cardiac autonomic health); **(3) Neurology** (#2 concussion); **(4) Psychology** (#3 anxiety, #4 biofeedback, #10 psychophysiological approach); and **(5) Physiology** (#5 endurance exercise, #12 left atrium, #13 parasympathetic reactivation). The layout reflects the structural relationships between these research hotspots.

As shown in the Figure 5, several key clusters were identified in the map, primarily including: #0 monitoring, #1 sleep, #2 concussion, #3 anxiety, #4 biofeedback, #5 endurance exercise, #6 fitness, #7indoor soccer, #8 court sports, #9 horse, #10 psychophysiological approach, #11 cardiac autonomic health, #12 left atrium, #13 parasympathetic reactivation, #14 correlations.

Based on the Timeline View, the major research foci for the application of heart rate variability in the sports field in recent years can be summarized as: Athlete Monitoring, Biofeedback, Sport-related Concussion, Anxiety, and Endurance Exercise. These five hot research directions will be analyzed in depth in the discussion section.

**Figure 6:**
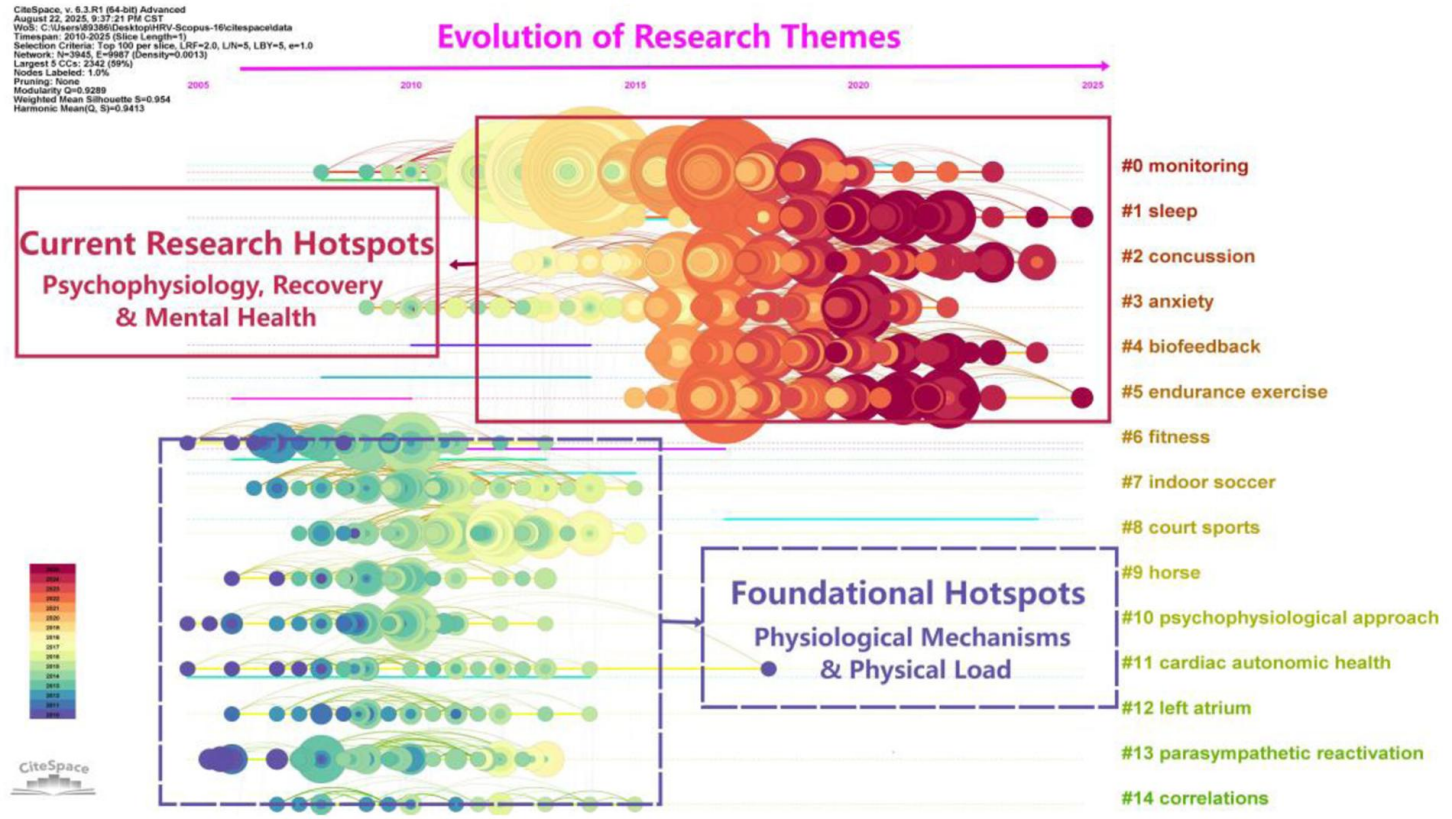
The timeline view of document co-citation clusters in heart rate variability in sports science. This visualization illustrates the evolutionary trajectory of research themes from 2010 to 2025. The horizontal axis represents the study timeline. Node size is proportional to citation frequency (influence), and node color indicates the publication time, showing a shift from foundational (cool colors) to current (warm colors) research activity.

As illustrated in Figure 6, this study constructed a timeline view of the knowledge evolution in the field of HRV in sports science using CiteSpace. This view not only reveals the core knowledge structure of the domain but also clearly delineates the emergence, development, and subsidence of various research hotspots (clusters) along a visual timeline. The overall network structure is well-defined, indicating that research in this field has entered a mature and diversified stage of development.

Structurally, the research content primarily revolves around three core domains: 1) fundamental physiological mechanisms, represented by “cardiac autonomic health” and “parasympathetic reactivation”; 2) applied exercise practices, centered on “endurance exercise” and “fitness”; and 3) athlete health and recovery, with a focus on “sleep,” “concussion,” and “anxiety.” Among these, “monitoring” serves as a methodological hub, bridging foundational theory with diverse application scenarios.

From the perspective of temporal evolution, the research trajectory in this field shows a clear trend of expansion from the exploration of basic physiological mechanisms toward diversified application scenarios. In recent years, the research frontier has significantly shifted towards utilizing HRV for fine-grained sleep quality assessment, post-concussion recovery tracking, and the quantitative evaluation of athletes’ psychological states (such as anxiety). This trend suggests that future research will increasingly focus on multi-dimensional integrated monitoring that incorporates physiological, psychological, and neural indicators to achieve personalized and precise management of athlete health and performance.

### 3.6 Analysis of keywords

Keywords are the essence and focus of an article, encapsulating its content and primary themes. In a specific field, the prominence of keywords, indicated by their co-occurrence frequency and centrality, reflects their importance as research hotspots.

**Figure 7:**
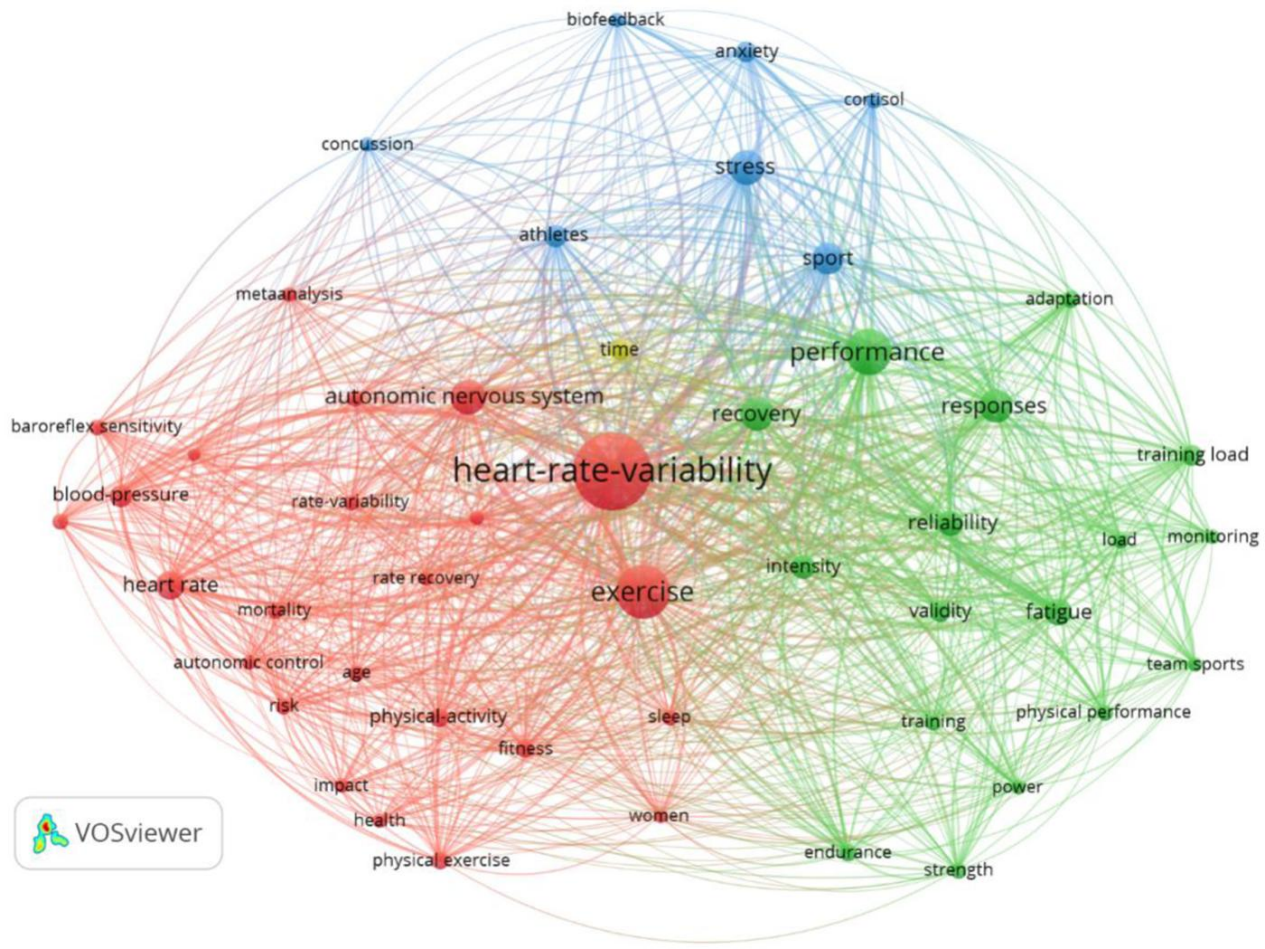
Keyword Co-occurrence network Map of research progress on heart rate variability in the sports domain.

Whereas the previous section's timeline view revealed the historical evolution of research hotspots from a longitudinal temporal dimension, this section provides a complementary analysis from a cross-sectional structural dimension. A keyword co-occurrence network offers a “snapshot” of the current intellectual landscape. As illustrated in Figure 7, this map visually represents the strength and proximity of connections between core themes, thereby outlining the field's overall knowledge framework.

The co-occurrence and centrality of keywords in a field reflect its research hotspots. In sports science (Figure 7), the nodes “exercise,” “autonomic nervous system,” “performance,” and “stress” form the research cornerstone alongside “heart rate variability” (HRV). Distinct clusters represent different research branches built upon this foundation.

Red Cluster (Physiological Basis): The node “exercise” acts as a critical bridge, linking HRV to fundamental mechanisms (“autonomic nervous system,” “baroreflex sensitivity”) and physiological indicators (“blood pressure,” “heart rate”). This cluster represents research on how exercise modulates HRV, reflecting its impact on autonomic function and health.

Green Cluster (Athletic Performance & Training): Centered on “performance,” this cluster links HRV directly to athletic outcomes. “Recovery” is a key intermediary node connecting the physiological state reflected by HRV with “training load” and “fatigue.” This highlights HRV's role in guiding personalized training and optimizing recovery.

Blue Cluster (Psychophysiological State): With “stress” as its central node, this cluster highlights HRV's use as a biomarker for the psychophysiological stress responses of “athletes.” The strong links between “stress,” HRV, “cortisol,” and “anxiety” underscore HRV's value in assessing psychophysical load and adaptation.

This map demonstrates that sports-related HRV research has evolved into distinct yet interconnected branches. The red cluster establishes the physiological basis, the green cluster details the instrumental value for performance, and the blue cluster reveals the importance for assessing psychophysiological states. Bridging keywords like “recovery” and “athletes” link these domains, forming a comprehensive picture of HRV's multifaceted applications in sports science and reflecting a developmental trend from fundamental mechanisms to applied practice.

**Figure 8:**
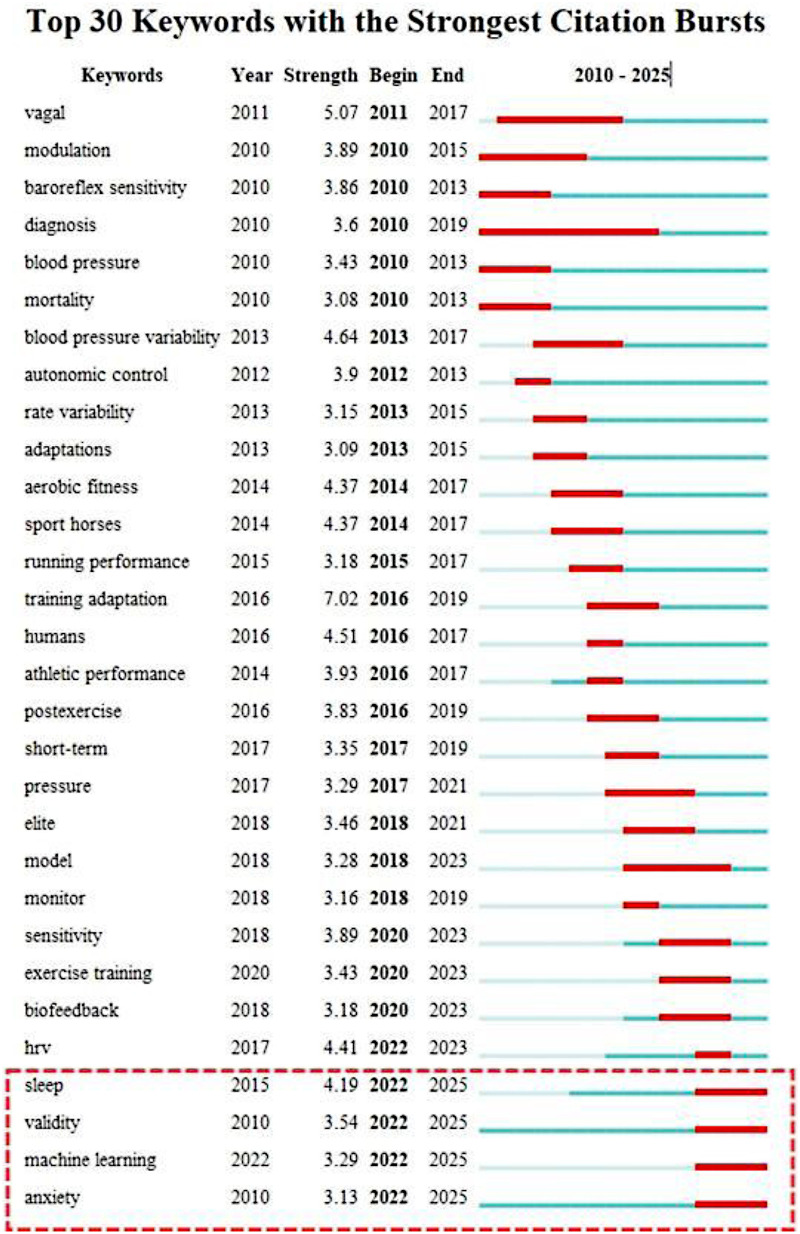
Top 30 keywords with the strongest citation bursts. The list displays keywords with significant surges in citation frequency. **Year:** Indicates the year the keyword first appeared in the dataset. **Strength:** Represents the intensity of the burst. **Timeline Visualization:** The **blue line** represents the **time interval of the keyword’s appearance** in the dataset. The **red segment** indicates the **active duration of the citation burst**, marking the specific period where the keyword received a sudden and significant increase in scholarly attention. The recent bursts for “Sleep,” “Machine Learning,” and “Anxiety” (extending to 2025) highlight current research frontiers.

Detecting keyword bursts helps to explore research hotspots with significant influence within a specific period. Changes in burst words can identify emerging frontier hotspots in the field. Figure 8 displays the keyword burst analysis from the Web of Science Core Collection database, showing the “Top 30 Keywords with the Strongest Citation Bursts.” In the figure, “Keywords” refers to the keywords themselves; “Year” indicates the year the keyword first appeared; “Strength” denotes the burst intensity; “Begin” is the year the burst started, and “End” is the year the burst concluded. As of 2025, the keywords exhibiting ongoing citation bursts are “Sleep,” “Machine Learning,” and “Anxiety”. An in-depth analysis of these three directions can provide a valuable reference for scholars in this field.

## 4 Discussion

### 4.1 Research hotspots analysis

#### 4.1.1 Athlete monitoring

HRV, as a non-invasive window for assessing ANS function, has been extensively studied for its effectiveness in monitoring training load and physiological stress. Clusters #1 (athlete monitoring) and #2 (photoplethysmography) in Figure 5 represent the research clusters formed around HRV concerning athlete monitoring.

Vagal-related metrics like RMSSD and HF power track training load (6). High-intensity training decreases HF power (30), while increased training load lowers morning resting RMSSD, which returns to baseline with recovery (4). Meta-analyses confirm that vagal indices such as RMSSD, HF, and SD1 reflect training adaptation (3). Positive adaptation, therefore, elevates resting and post-exercise HRV and accelerates heart rate recovery (HRR), making HRV a key monitoring tool.

Longitudinal monitoring is key. A sustained drop below an individual's baseline resting HRV signals accumulated stress and overtraining risk (10). This objective tracking of internal states enables more precise training regulation than subjective reports (1). Given significant individual responses to training (31), HRV-guided training more effectively improves endurance performance than standardized plans (8).

However, HRV application faces challenges. Non-training factors like sleep, mood, and nutrition can confound interpretation (10). Critically, both positive adaptation and functional overreaching (FOR) can paradoxically increase post-exercise HRV and accelerate HRR, complicating their differentiation (3). Accurate assessment thus requires contextual data on training load and performance. The effect of FOR on resting HRV can be minor or inconsistent, making measurement timing crucial (3). Further research is needed to standardize metrics, data collection protocols, and individualized thresholds.

In summary, HRV is a potent tool for monitoring athletic training response, especially for personalizing training and preventing overtraining. Effective application, however, demands individualized baselines, control for confounding variables, and careful interpretation within the broader training context.

#### 4.1.2 HRV biofeedback

Heart rate variability biofeedback (HRV-BF) is a technique aimed at enhancing the self-regulatory capacity of ANS; in Figure 5, cluster #4 is formed by literature concerning HRV biofeedback. The importance of HRV-BF lies in its provision of a non-pharmacological means to help athletes learn how to actively regulate their physiological state, thereby optimizing performance under high-pressure environments, improving emotional control, and potentially promoting recovery (32, 33). This technique guides individuals to maximize HRV through specific exercises (primarily slow, rhythmic breathing) by providing real-time HRV information feedback, consequently enhancing the adaptability and resilience of the cardiovascular system.

Through HRV-BF training, athletes can learn to enter a state of “physiological coherence,” characterized by smooth, sine-wave-like heart rate patterns with high amplitude at a specific frequency (typically the individual's resonant breathing frequency, around 0.1 Hz or 6 breaths/minute) (33). Systematic reviews and empirical studies indicate that this training may yield multifaceted positive effects for athletes. Evidence suggests that HRV-BF might contribute to enhancing performance in specific motor skills, particularly in tasks requiring precise control and psychological stability (e.g., shooting, golf), although the magnitude and universality of these effects necessitate further high-quality research (34, 35). Furthermore, HRV-BF is widely recognized for its effectiveness in helping athletes manage competitive anxiety by enhancing vagal tone to regulate emotional responses (36, 37).

The practical application of HRV-BF typically involves monitoring heart rate using sensors (such as finger-clip or chest-strap heart rate monitors) and visualizing HRV indicators (e.g., heart rate waveform, power spectral density, or coherence scores) for the user via software. The core of the training is to guide users in performing slow, steady breathing at a specific frequency while observing the feedback signals, thereby learning how to actively increase HRV amplitude and rhythmicity through respiration (33, 38). Training effects may also be associated with other psychological skills, such as improved concentration and subjective experiences of coping with stress. Research has also compared the effects of HRV-BF with other biofeedback techniques (e.g., electromyographic biofeedback) on task performance, suggesting its potential unique advantages in certain cognitive or motor tasks (39).

Based on current understanding, HRV-BF offers athletes a promising tool for managing pre-competition anxiety, stabilizing physiological states during critical moments, and possibly promoting recovery. However, its effective implementation necessitates professional guidance, regular practice, and active participation from the athlete to achieve desired outcomes (32).

#### 4.1.3 Sport-related concussion

HRV, as a potential tool for the assessment and recovery monitoring of sport-related concussion (SRC), has become one of the research hotspots in this field. Cluster #3 in Figure 5 is formed by literature related to HRV and sport-related concussion.

The objective assessment and management of sport-related concussion (SRC) are ongoing challenges in sports medicine. HRV, as a non-invasive measure of ANS function, is gaining widespread attention due to its ability to reflect the common ANS dysfunction observed following SRC. By measuring HRV, objective physiological information regarding the state of the autonomic nervous system can be obtained, which offers a perspective that complements subjective symptom reports in assessing concussion recovery (40).

SRC typically leads to an ANS imbalance, characterized by a relative increase in sympathetic activity and/or a decrease in parasympathetic (vagal) activity; these changes can be captured through HRV measurements. Multiple lines of research evidence indicate that, compared to healthy control groups or individual baselines, athletes in the acute phase of SRC commonly exhibit a significant reduction in HRV, particularly in indices reflecting vagal modulation (such as RMSSD and HF power) (41, 42). This suppressed state of HRV often persists while the athlete's symptoms continue and gradually returns to normal with the alleviation of clinical symptoms and the restoration of physiological function (42). Persistent HRV suppression may predict a longer recovery period or protracted symptoms. Furthermore, existing research has linked low post-SRC HRV to a more severe symptom burden (e.g., headache, dizziness, cognitive difficulties) and declines in cognitive function performance (40, 43–45).

The assessment of ANS function after SRC is not limited to resting HRV; dynamic assessments (such as HRV responses to postural changes or standardized low-intensity exercise) also show potential and may be more capable of revealing latent ANS reactivity abnormalities, such as exercise intolerance (46–48). Commonly used assessment indicators include RMSSD, HF, LF, and the LF/HF ratio. The mechanisms underlying ANS dysfunction post-SRC are complex and may involve damage to central autonomic networks, neuroinflammation, or alterations in baroreflex sensitivity (40).

Although HRV shows significant potential in SRC assessment, realizing its clinical value still requires addressing key issues. Future priority directions include: validating the clinical utility of dynamic/stress-induced HRV assessment in guiding “return-to-play” decisions and other aspects (48, 49); deeply investigating the underlying mechanisms driving persistent autonomic dysfunction after SRC (50); and, crucially, advancing the standardization of measurement protocols and interpretation criteria to overcome barriers to clinical translation and ensure the reliability and comparability of results.

#### 4.1.4 Endurance exercise

Heart rate variability has become a core biomarker for quantifying adaptations to endurance training (51). Long-term endurance training can remodel the autonomic regulation of the heart, a process typically characterized by enhanced parasympathetic (vagal) tone and attenuated sympathetic activity, ultimately manifesting as a lower resting heart rate (training-induced bradycardia) and an increase in overall HRV levels (52, 53). This adaptation stems not only from changes in neural regulation but also involves the remodeling of the intrinsic electrophysiological properties of the sinoatrial node. For instance, research has found that exercise training can reduce intrinsic heart rate by down-regulating HCN4 ion channels (the “funny” channels) (54).

In recent years, HRV-guided training has shown great potential for individualizing training programs. Studies have demonstrated that dynamically adjusting training intensity based on daily HRV data, compared to following a predefined training plan, can more effectively improve an athlete's maximal oxygen uptake (VO_2_max) and performance, while reducing the proportion of non-responders to training (55). The success of this approach relies on the precise interpretation of HRV data, which requires consideration of various factors such as sex (females often exhibit higher parasympathetic modulation) (52, 56), age, and non-training-related stressors like sleep and psychological stress (57).

Methodological rigor is fundamental to the application of HRV. Although single-day HRV values can fluctuate significantly, using methods like a 7-day rolling average can more reliably track trends in autonomic nervous system changes induced by training (58). With technological advancements, ultra-short-term (e.g., 1 min) HRV measurements using smartphone applications and chest straps have been validated as both effective and reliable, greatly promoting the application of HRV in real-world training environments (59, 60). In summary, HRV is not only a profound reflection of the physiological adaptations to endurance training but also a powerful tool for achieving a more scientific and individualized approach to training management.

### 4.2 Emerging trends

#### 4.2.1 Anxiety

As illustrated in Figures 5, 8, the role of HRV as a biomarker for athlete anxiety in the sports domain is both a current research hotspot and a future research trend. Since the literature search commencement in 2010, studies concerning the relationship between heart rate variability and anxiety in athletes have garnered widespread attention. A significant surge in citations occurred in 2022 and has continued into 2025, establishing this as an ongoing and future research trajectory.

The neurophysiological basis of anxiety is intimately linked with ANS activity. Extensive research and meta-analyses have confirmed that lower resting HRV is associated with higher trait anxiety and an increased risk of anxiety disorders (61, 62). Cardiac autonomic regulation is considered a crucial physiological foundation for emotional regulation capacity. Consequently, the potential of HRV as a biomarker for athlete anxiety has attracted considerable attention in the sports field.

Studies involving athletes have provided some supporting evidence. Certain research has observed that athletes with high pre-competition anxiety exhibit suppressed vagal activity (63). Concurrently, physical exercise, such as resistance training (64) and mind-body practices like Tai Chi and Yoga (65), can alleviate anxiety by improving ANS function (i.e., increasing HRV). Various forms of training have been demonstrated to enhance HRV in healthy individuals (66), thereby offering a pathway for exercise interventions in anxiety.

However, the complexity of the relationship between HRV and anxiety in athletes is a current research hotspot. A recent meta-analysis focusing on athletes indicated that the direct association between HRV (as represented by RMSSD) and multiple subjective psychophysiological factors (including stress, mood, and fatigue, which are closely related to anxiety) is not significant (62). This suggests that within populations of trained athletes, the relationship between HRV and subjective anxiety is not a simple negative correlation. Instead, it appears to be intricately modulated by a multitude of factors, including training status, adaptation level, coping strategies, and physiological resilience. Furthermore, studies utilizing wearable devices in naturalistic settings have also found that the strength of the association between HRV and perceived stress/anxiety is weaker than that observed in laboratory environments (67), underscoring the complexities of real-world conditions and the limitations of current measurement technologies.

Future research must extend beyond simple correlational analyses to delve deeper into the influence of moderating factors, such as training load and psychological skills, on the HRV-anxiety relationship in athletes. Investigations should pivot towards dynamic process analysis, examining the trajectory of HRV changes in specific contexts, such as pre-competition periods. Moreover, an emphasis on methodological rigor is crucial, encompassing the adoption of standardized measurement protocols, control of confounding variables, and validation of the reliability and validity of different technologies in authentic sporting environments (62, 67).

#### 4.2.2 Machine learning

As shown in Figure 8, research in this field has seen a significant increase in the application of machine learning (ML) since 2022. ML offers powerful tools to overcome the inherent complexities and significant individual differences in HRV analysis (68), rapidly becoming a research hotspot and a future development direction.

ML algorithms significantly enhance the predictive efficacy and application scope of HRV data by processing high-dimensional, complex datasets, identifying patterns that are difficult for traditional statistics to capture, and enabling personalized assessments. ML can utilize HRV data to predict key physical fitness indicators, such as maximal oxygen uptake (VO_2_max) (69) and aerobic/anaerobic thresholds (70), providing athletes with convenient physiological assessment methods. Furthermore, by integrating HRV with multidimensional data such as training load and sleep, ML models can effectively predict an athlete's perceived recovery state (71) and mental health status (e.g., stress, anxiety, depression) (72).

To provide a structured technical overview of this emerging trend, we have summarized the mainstream algorithms and their specific applications identified in our bibliometric analysis. As presented in Table 4, the field is evolving from basic physiological estimation using regression models to complex risk prediction and psychophysiological state monitoring using ensemble and deep learning architectures.

**Table 4:** Mainstream machine learning algorithms for HRV analysis.

Model type	Specific algorithms	Key applications	Representative citations
Regression	Linear, Lasso	Estimating physiological metrics (e.g., VO2max)	(69)
Classification	SVM, Decision Trees	Identifying athlete profiles, fatigue, and mental workload	(73, 88, 89)
Ensemble	Random Forest, XGBoost	Predicting injury risk and daily recovery status	(90, 71, 91)
Deep Learning	CNN, LSTM	Modeling complex stress patterns and mood states	(92, 93, 72)

SVM, support vector machine; RF, random forest; CNN, convolutional neural networks; LSTM, long short-term memory; VO_2max_, maximal oxygen uptake.

Beyond specific algorithms, the transformative value of ML lies in its capacity for non-linear modeling and personalization. Unlike linear methods, ML models effectively capture the complex interactions between multiple physiological stressors (73, 74). Crucially, ML facilitates the creation of highly personalized models. Research has shown that the predictive accuracy of individualized models far surpasses that of group models, and the most predictive combination of indicators varies among different athletes (71). This indicates a trend towards using ML to establish customized monitoring and prediction models for each athlete to achieve truly individualized training guidance (71, 74).

In summary, the fusion of ML and wearable technology is revolutionizing the application of HRV in sports science, pushing it towards greater precision and individualization to optimize training strategies, enhance athletic performance, and safeguard athletes’ health (75). Despite its promising prospects, future research must continue to address issues such as data interpretation, model robustness, and scalability in practical applications (76).

#### 4.2.3 Sleep quality

Research on exercise and sleep has grown explosively since 2022 (Figure 8), as sleep quality is critical for athlete recovery and performance (77, 78). Insufficient sleep impairs performance and increases injury risk (79), yet it is prevalent among athletes, whose average total sleep time is only 7.2 h with suboptimal efficiency (80, 81).

Exercise's impact on sleep depends on its timing and intensity; moderate exercise generally improves sleep, while high-intensity exercise near bedtime can be disruptive (82). This effect is mediated by ANS regulation, reflected in HRV dynamics. While long-term training enhances resting and nocturnal HRV (a reliable metric with ICC >0.9), acute exercise temporarily suppresses it (11). Specifically, impaired vagal reactivation after evening high-intensity exercise is a primary physiological pathway to sleep disturbances (83).

However, this relationship is not universal and is highly context-dependent. For instance, in elite female soccer players, no significant link was found between training load and nocturnal HRV, suggesting HRV's sensitivity as a monitoring tool may be lower in intermittent team sports compared to endurance sports (84). Further complicating interpretation, a meta-analysis found that both positive adaptation and functional overreaching can paradoxically increase vagal-related HRV and post-exercise heart rate recovery (HRR), while resting-state HRV may be insensitive to overtraining (3). The influence of training load on nocturnal HRV is therefore neither linear nor universal, but is modulated by sport type, training phase, and individual differences.

Given the reliability of nocturnal HRV and the complexity of its response, future research must examine the precise relationship between sleep microarchitecture (e.g., SWS) and HRV dynamics under different training stresses (85, 86). Developing advanced algorithms that integrate multi-source data—including sleep staging, training load, and subjective reports—is crucial for achieving personalized and accurate HRV interpretation (87).

### 4.3 Limitations

While this study provides a comprehensive bibliometric analysis, several inherent limitations must be acknowledged. First, data retrieval was restricted to the Web of Science Core Collection and Scopus databases. Although these are authoritative sources, this selection criteria may have excluded relevant studies indexed solely in other databases (e.g., PubMed, Embase) or grey literature, potentially leading to coverage bias.

Second, the analysis was limited to English-language publications to ensure the accuracy of keyword co-occurrence and clustering algorithms in CiteSpace and VOSviewer. This restriction may result in the omission of significant contributions published in other languages, particularly from regions with strong sports science traditions.

Third, bibliometric indicators are subject to a “citation lag” phenomenon. Recently published high-quality studies, especially those from 2024 to 2025, may not yet have accrued sufficient citations to appear as major nodes or bursts in the visualization maps, despite their potential future impact. Finally, it is important to note that the data for 2025 is partial. Therefore, the bibliometric trends for the current year represent an incomplete dataset rather than a decline in research interest or output.

## 5 Conclusion

This study conducted a comprehensive scientometric analysis of the literature on HRV applications in sports science from 2010 to early 2025. The research volume in this field has shown a significant growth trend, peaking in 2022. Nakamura, Fabio Y. was identified as the most prolific scholar, while CAMM AJ was the most co-cited author. At the national level, the United States and Brazil had the highest output, and at the institutional level, the University of São Paulo was the leader. The document co-citation cluster analysis revealed five major research hotspots: Athlete Monitoring, Biofeedback, Sport-related Concussion, Anxiety, and Endurance Exercise. Furthermore, keyword burst analysis identified three frontier research directions that represent current and future trends: “Sleep,” “Machine Learning,” and “Anxiety.”

Practically, these findings highlight a clear shift in how HRV is used in sports. It is no longer just a tool for monitoring training load; it is now essential for tracking sleep quality and managing psychological stress. Furthermore, the rapid growth of machine learning offers a new way to analyze complex physiological data, allowing coaches and scientists to move beyond general guidelines and develop truly individualized training plans for each athlete.

In summary, this study provides a macroscopic perspective on the knowledge structure and developmental dynamics of HRV research in sports. By identifying key contributors, major research hotspots, and emerging trends, it offers a clear roadmap and valuable reference for future research in this domain.

## Data Availability

The original contributions presented in the study are included in the article/Supplementary Material, further inquiries can be directed to the corresponding author.
